# An On-Demand Charging for Connected Target Coverage in WRSNs Using Fuzzy Logic and Q-Learning †

**DOI:** 10.3390/s21165520

**Published:** 2021-08-17

**Authors:** Phi Le Nguyen, Van Quan La, Anh Duy Nguyen, Thanh Hung Nguyen, Kien Nguyen

**Affiliations:** 1 The School of Information and Communication Technology, Hanoi University of Science and Technology, Ha Noi 11615, Vietnam; quan.lv133135@sis.hust.edu.vn (V.Q.L.); duy.na184249@sis.hust.edu.vn (A.D.N.); hungnt@soict.hust.edu.vn (T.H.N.); 2 The Graduate School of Engineering, Chiba University, 1-33, Yayoi-cho, Inage-ku, Chiba 263-8522, Japan; nguyen@chiba-u.jp

**Keywords:** WRSN, Q-learning, on-demand charging algorithm, target coverage, connectivity

## Abstract

In wireless rechargeable sensor networks (WRSNs), a mobile charger (MC) moves around to compensate for sensor nodes’ energy via a wireless medium. In such a context, designing a charging strategy that optimally prolongs the network lifetime is challenging. This work aims to solve the challenges by introducing a novel, on-demand charging algorithm for MC that attempts to maximize the network lifetime, where the term “network lifetime” is defined by the interval from when the network starts till the first target is not monitored by any sensor. The algorithm, named Fuzzy Q-charging, optimizes both the time and location in which the MC performs its charging tasks. Fuzzy Q-charging uses Fuzzy logic to determine the optimal charging-energy amounts for sensors. From that, we propose a method to find the optimal charging time at each charging location. Fuzzy Q-charging leverages Q-learning to determine the next charging location for maximizing the network lifetime. To this end, Q-charging prioritizes the sensor nodes following their roles and selects a suitable charging location where MC provides sufficient power for the prioritized sensors. We have extensively evaluated the effectiveness of Fuzzy Q-charging in comparison to the related works. The evaluation results show that Fuzzy Q-charging outperforms the others. First, Fuzzy Q-charging can guarantee an infinite lifetime in the WSRNs, which have a sufficient large sensor number or a commensurate target number. Second, in other cases, Fuzzy Q-charging can extend the time until the first target is not monitored by 6.8 times on average and 33.9 times in the best case, compared to existing algorithms.

## 1. Introduction

Wireless Sensor Networks (WSNs) have found various applications, such as air quality monitoring, environmental management, etc., [1,2]. A WSN typically includes many battery-powered sensor nodes, monitoring several targets, and sending sensed data to a base station for further processing. In the WSNs, it is necessary to provide sufficient monitoring quality surrounding the targets (i.e., guaranteeing target coverage). Moreover, the WSNs need to have an adequate capacity for the communication between the sensors and base station (i.e., ensuring connectivity) [3,4,5]. The target coverage and connectivity are severely affected by the depletion of the battery on sensor nodes. When a node runs out of battery, it becomes a dead node without sensing and communication capability, damaging the whole network in consequence. Wireless Rechargeable Sensor Networks (WRSNs) leverages the advantages of wireless power transferring technology to solve that critical issue in WSNs. A WRSN uses a mobile charger (MC) to wirelessly compensate for a rechargeable battery’s energy consumption on a sensor node, aiming to guarantee both the target coverage and connectivity.

In a normal operation, the MC moves around the networks and performs charging strategies, which can be classified into periodic [6,7,8,9,10] or on-demand charging [11,12,13,14,15,16,17]. In the former, the MC, with a predefined trajectory, stops at charging locations to charge the nearby sensors’ batteries. In the latter, the MC will move and charge upon receiving requests from the sensors, which have the remaining energy below a threshold. The periodic strategy is limited since it cannot adapt to the sensors’ energy consumption rate dynamic. On the contrary, the on-demand charging approach potentially deals with the uncertainty of the energy consumption rate. Since a sensor with a draining battery triggers the on-demand operation, the MC’s charging strategy faces a new time constraint challenge. The MC needs to handle two crucial issues: deciding the next charging location and the staying period at the location.

Although there are many, the existing on-demand charging schemes in the literature face two serious problems. The first one is the consideration of the same role for the sensor nodes in WRSNs. That is somewhat unrealistic since, intuitively, several sensors, depending on their locations, significantly impact the target coverage and the connectivity than others. Hence, the existing charging schemes may enrich unnecessary sensors’ power while letting necessary ones run out of energy, leading to charging algorithms’ inefficiency. It is of great importance to take into account the target coverage and connectivity simultaneously. The second problem is about the MC’s charging amount, which is either a full capacity (of sensor battery) or a fixed amount of energy. The former case may cause: (1) a long waiting time of other sensors staying near the charging location; (2) quick exhaustion of the MC’s energy. In contrast, charging a too small amount to a node may lead to its lack of power to operate until the next charging round. Therefore, the charging strategy should adjust the transferred energy level dynamically following the network condition.

Motivated by the above, we propose a novel on-demand charging scheme for WRSN that assures the target coverage and connectivity and adjusts the energy level charged to the sensors dynamically. Our proposal, named Fuzzy Q-charging, aims to maximize the network lifetime, which is the period from when the network starts till the first target is not monitored by any sensor. A target is considered to be monitored by a sensor if it is covered by a sensor (i.e., remaining within the sensor’s sensing range) and the sensor is connected to the base station (i.e., a routing path exists between the sensor and the base station). To achieve this ultimate goal, Fuzzy Q-charging attempts to extend the lifetime of the sensors, in which, sensors that contribute more to target monitoring will be prioritized more. Fuzzy Q-charging combines two techniques: Fuzzy logic and Q-learning, each of which is designed to accomplish a given task with a certain goal. First, we exploit Fuzzy logic in an optimization algorithm that determines the optimal charging time at each charging location. The Fuzzy logic-based algorithm aims at maximizing the number of alive sensors. Fuzzy logic is used to cope with network dynamics by taking various network parameters into account during the determination process of the optimal charging time. Second, given the optimal charging time at every charging location, we leverage the Q-learning technique to select the next charging location to maximize the network lifetime. The MC maintains a Q-table containing the charging locations’ Q-values representing the charging locations’ goodness. The Q-values will be updated in a real-time manner whenever there is a new charging request from a sensor. We design the Q-value to prioritize charging locations at which the MC can charge a node depending on its critical role. After finishing tasks in one place, the MC chooses the next one, which has the highest Q-value, and determines an optimal charging time. The main contributions of the paper are as follows.

- We propose a Fuzzy logic-based algorithm that determines the energy level to be charged to the sensors. The energy level is adjusted dynamically following the network condition.
- Based on the above algorithm, we introduce a new method that optimizes the optimal charging time at each charging location. It considers several parameters (i.e., remaining energy, energy consumption rate, sensor-to-charging location’s distance) to maximize the number of alive sensors.
- We propose *Fuzzy Q-charging*, which uses Q-learning in its charging scheme to guarantee the target coverage and connectivity. *Fuzzy Q-charging*’s reward function is designed to maximize the charged amount to essential sensors and the number of monitored targets.

The remainder of the paper is constructed as follows. Section 3 describes the network model and Q-learning. We briefly review the related work in Section 2. Section 4 introduces our proposed algorithm. Section 5 includes the performance evaluation. Finally, Section 6 concludes the paper and shows our future work.

## 2. Related Work

Initially, we introduce the existing works related to periodic charging in WRSNs. In [6], the authors leverage PSO and GA to propose a charging path determination algorithm that minimizes the docking time during which the MC recharges itself at the depot. Ref. [7] jointly considers charging path planning and depot positioning to minimize the number of MCs while ensuring no sensor runs out of energy before being recharged. The work in [8] determines a charging path to maximize the MC’s accumulative charging utility gain or minimize the MC’s energy consumption during traveling. The authors then propose approximation algorithms with constant ratios for the maximization and minimization problems. Arguing that an MC can not fulfill all sensors’ demand in dense networks, W. Xu et al. in [9] introduce a multi-chargers approximation model to increase the charging speed. In [10], C. Lin et al. derive a new energy transfer model with distance and angle factors. They also consider the problem of minimizing the total charging delay for all nodes. They use linear programming and obtain the optimal solution. As the charging schedule is always fixed, the periodic scheme fails to adapt to the dynamic of sensors’ energy consumption.

Regarding the on-demand charging, the authors in [16] address the node failure problem. They first propose to choose the next charging node based on the charging probability. Second, they introduce a charging node selected method to minimize the number of other requesting nodes suffering from energy depletion. In [12,13], aiming to maximize the charging throughput, they propose a double warning threshold charging scheme. Two dynamic warning thresholds are triggered depending on the residual energy of sensors. The authors in [17] studied how to optimize the serving order of the charging requests waiting in the queue using the gravitational search algorithm. In [15], X. Cao et al. introduce a new metric (i.e., charging reward), which quantifies the charging scheme’s quality. The authors then address the problem of maximizing the total reward in each charging tour under the constraint of the MC’s energy and sensors’ charging time windows. They use a deep reinforcement learning-based on-demand charging algorithm to solve the addressed problem.

The existing charging algorithms have two serious problems. First, the charging time problem has not been thoroughly considered. Most of the charging schemes leverage either the fully charging approach [6,7,8,11,12,13,16] or the partial charging one [18]. We want to emphasize that the charging time is an essential factor that decides how much the charging algorithm can prolong the network lifetime. Moreover, there is no existing work considering the target coverage and connectivity constraints concurrently. Most previous works treat all sensors in WRSNs evenly; hence, the MC may charge unnecessary sensors while necessary ones may run out of energy. Unlike them, this work addresses the target coverage and connectivity constraints in charging schedule optimization. We uniquely consider the optimization of charging time and charging location simultaneously. We use Fuzzy logic and Q-learning in our proposal.

Fuzzy logic has been applied in many fields, such as signal processing [19,20], robotics [21], and embedded controllers [22]. In WSNs, Fuzzy logic is a promising technique in dealing with various problems, including localization, routing [23,24], clustering [25], and data aggregation [26,27]. R. M. Al-Kiyumi et al. in [23] propose a Fuzzy logic-based routing for lifetime enhancement in WSNs, which maps the network status into corresponding cost values to calculate the shortest path. In [28], the authors also leverage Fuzzy logic and Q-learning but in a cooperative multi-agent system for controlling the energy of a microgrid. In [29], Fuzzy and Q-learning are combined to address the problem of thermal unit commitment. Specifically, each input state vector is mapped with the Fuzzy rules to determine all the possible actions with the corresponding Q-values. The main idea is exploiting Fuzzy logic to map the network status into corresponding cost values to calculate the shortest path. Recently, the authors in [14] use Fuzzy logic in an algorithm for adaptively determining the charging threshold and deciding the charging schedule. Different from the others, we use Fuzzy logic and Q-learning in our unique Fuzzy Q-charging proposal. The earlier version of this work has been published in [30], which considers only Q-charging.

## 3. Network Model, Q-Learning, and Fuzzy Logic

### 3.1. Network Model and Problem Definition

**Figure 1:**
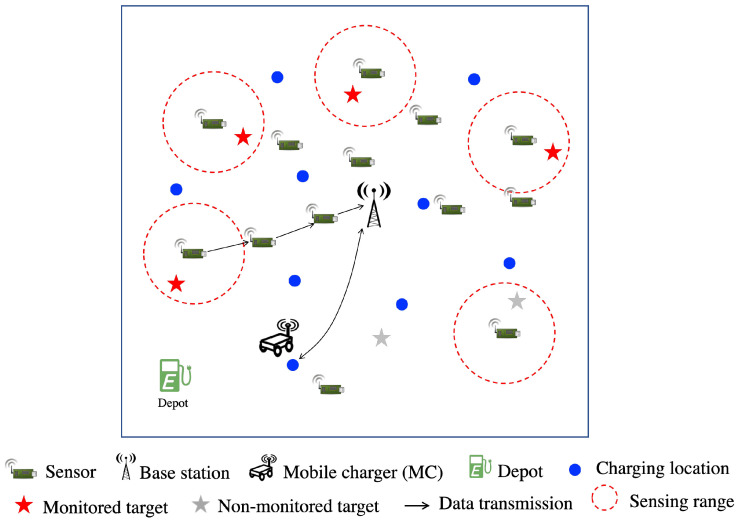
The network model.

Figure 1 shows the considered network model, in which a WRSN monitors several targets. The network has three main components: an MC, sensor nodes, and a base station. The MC is a robot that can move and carry a wireless power charger. The sensor nodes can receive charged energy from the MC via a wireless medium. The base station is static and responsible for gathering sensing information. We assume that there are *n* sensors $S_{j}$ (j=1,…,n) and *m* targets $T_{k}$ (k=1,…,m). We call a sensor a *target-covering* sensor if it covers at least one target. Moreover, if there exists an alive routing path between a sensor and the base station, it is *connected* to the base station. The target is defined as to be *monitored* when at least one sensor connected to the base station covers it.

A sensor node that has its remaining energy below $E_{th}$ (i.e., a predefined threshold) will send a charging request to the base station. The base station then uses one-hop routing to transfer the request to the MC. We assume that the MC can interact with the base station over a long-range communication. We target a non-preemptive charging schedule, in which charging requests from sensors are queued at the MC. We assume that there are *k* charging locations denoted by $D_{1},\ldots ,D_{k}$ in the network. When the MC completes its tasks at a charging location, it runs our proposed algorithm to select the next optimal charging location from $D_{1},\ldots ,D_{k}$. Moreover, the MC also determines the optimal charging time at that charging location. When the energy of the MC goes below a threshold, it returns to the depot to recharge itself. Besides gathering the sensing information, the base station is also responsible for collecting information about the remaining energy sensors. Based on that, the MC estimates every sensor’s energy consumption rate using the weighted averaging method. Given all sensors and the targets’ locations, our on-demand charging algorithm aims to maximize the network lifetime.

### 3.2. Q-Learning

**Figure 2:**
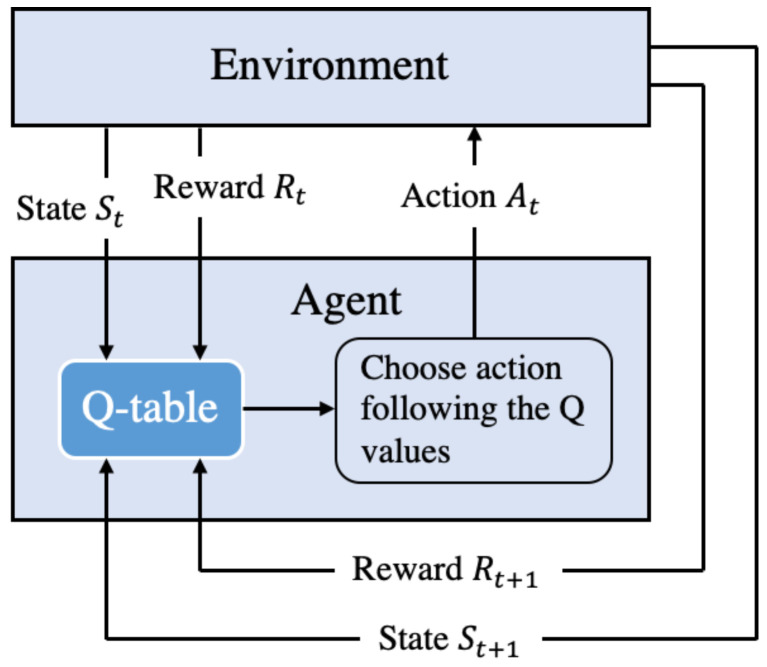
Q-learning overview.

Q-learning is a reinforcement learning technique that is widely used in making a decision. The main idea is to achieve a specific goal based on experience learning from the past. The standard Q-learning framework consists of four components: an environment, one or more agents, a state space, and an action space, as shown in Figure 2. The Q-value represents the approximate goodness of the action concerning the agent’s goal. An agent chooses actions according to the policy and the Q-value. After performing an action, the agent modifies its policy to attain its goal. The Q-value is updated using the Bellman equation as follows:

(1) $$Q\left(S_{t},A_{t}\right)\leftarrow \left(1-\alpha \right)Q\left(S_{t},A_{t}\right)+\alpha \left[R_{t}+\gamma \;\underset{a}{max}\;Q\left(S_{t+1},a\right)\right],$$

where $Q(S_{t},A_{t})$ is the Q-value of action $A_{t}$ at a given sate $S_{t}$. $R_{t}$ is the reward obtained if performing action $A_{t}$ in the state $S_{t}$. Moreover, $\underset{a}{max}\;Q\left(S_{t+1},a\right)$ is the maximum possible Q-value in the next state $S_{t+1}$ for all possible actions *a*. α and γ are the learning rate and the future reward discount factor. Their values are set between 0 and 1.

### 3.3. Fuzzy Logic

A fuzzy logic system consists of three components: *fuzzification*, *fuzzy logic controller*, and *defuzzification*. The first component converts the crisp values of the variable into their fuzzy form using some membership functions. The second one is responsible for simulating the human reasoning process by making *fuzzy inference* based on inputs and a set of defined **IF-THEN** rules. The module itself can be separated into two subcomponents, namely *Knowledge Base* and *Inference Engine*. *Knowledge Base* is a set of specifically designed rules so that together with the input states of variables, they will produce consistent results. Each rule’s form is “**IF** {set of input} **THEN** {output}”. More explicitly, a fuzzy rule $R_{i}$ with *k*-inputs and 1-output has the following form.

(2) $$\begin{array}{cc} R_{i}: & \mathrm{IF}\left(I_{1}\;\mathrm{is}\;A_{i1}\right)\Theta \left(I_{2}\;\mathrm{is}\;A_{i2}\right)\Theta \ldots \Theta \left(I_{k}\;\mathrm{is}\;A_{ik}\right)\\ \; & \mathrm{THEN}\;(O\;\mathrm{is}\;B_{i}), \end{array}$$

where $\left\{I_{1},\ldots ,I_{k}\right\}$ represents the crisp inputs to the rule. $\left\{A_{i1},\ldots ,A_{ik}\right\}$ and $B_{i}$ are linguistic variables. The operator Θ can be **AND**, **OR**, or **NOT**. The *Inference Engine* is in charge of the estimation of the Fuzzy output set. It calculates the membership degree (μ) of the output for all linguistic variables by applying the rule set described in the *Knowledge Base*. For Fuzzy rules with lots of inputs, the output calculation depends on the operators used inside it, i.e., **AND**, **OR**, or **NOT**. The calculation for each type of operator is described as follows:

$$\begin{array}{cc} (I_{i}\;\mathrm{is}\;A_{i}\mathrm{AND}I_{j}\;\mathrm{is}\;A_{j}):\\ \mu_{A_{i}\cap A_{j}}\left(I_{ij}\right) & =\min\left(\mu_{A_{i}}\left(I_{i}\right),\mu_{A_{j}}\left(I_{j}\right)\right),\\ (I_{i}\;\mathrm{is}\;A_{i}\mathrm{OR}I_{j}\;\mathrm{is}\;A_{j}):\\ \mu_{A_{i}\cup A_{j}}\left(I_{ij}\right) & =\max\left(\mu_{A_{i}}\left(I_{i}\right),\mu_{A_{j}}\left(I_{j}\right)\right),\\ (\mathrm{NOT}I_{i}\;\mathrm{is}\;A_{i}):\\ \mu_{\bar{A_{i}}}\left(I_{i}\right) & =1-\mu_{A_{i}}\left(I_{i}\right). \end{array}$$

The last component helps to convert the fuzzy output set from the linguistic variables into a crisp value. The most popular fuzzy solution is a methodology called the centroid technique, described as follows:

(3) $$\mathrm{Center}\;\mathrm{of}\;\mathrm{Gravity}\;\mathrm{of}\;B\left({\mathrm{CoG}}_{B}\right)=\frac{\int_{-\infty }^{+\infty }\mu_{B}\left(z\right)zdz}{\int_{-\infty }^{+\infty }\mu_{B}\left(z\right)dz},$$

where $\mu_{B}\left(z\right)$ is the output membership function of the linguistic variable *B*.

## 4. Fuzzy Q-Charging Algorithm

### 4.1. Overview

We follow the on-demand charging strategy, in which a sensor sends a charging request to the base station when its energy is below a predefined threshold $E_{th}$. The base station then uses one-hop routing to transfer the request to the MC. The request is inserted into the waiting list at the MC. The MC then performs the following procedures to update the Q-table:

- The MC leverages Fuzzy logic to calculate a so-called *safe energy level* (denoted as $E_{sf}$), which is sufficiently higher than $E_{th}$. The MC then uses the algorithm described in Section 4.3 to determine the charging time at each charging location. The charging time is optimized to maximize the number of sensors that guarantee the safe energy level.
- The MC calculates the reward of every charging location using Equation (12) and updates the Q-table using Equation (4).

**Figure 3:**
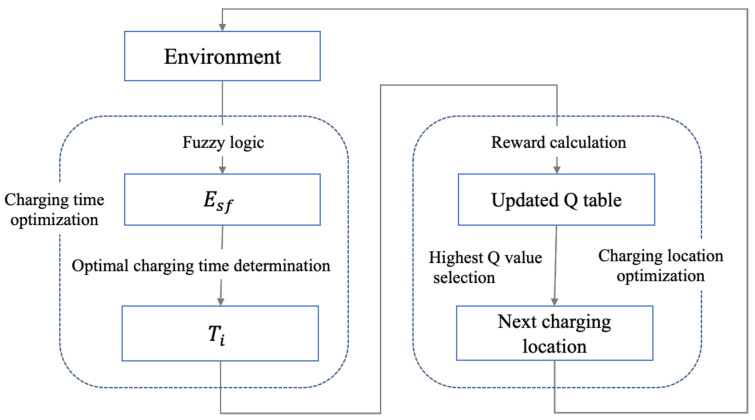
The flow of Fuzzy Q-learning-based charging algorithm.

After finishing charging at a charging location, the MC selects the next charging location as the one with the highest Q-value. Finally, the MC moves to the next charging location and charges for the determined charging time. When the energy of the MC goes below a threshold, it returns to the depot to recharge itself. Figure 3 presents the overview of our charging algorithm. To facilitate the reading, we summarize all the used notations in Table 1.

### 4.2. State Space, Action Space and Q Table

**Figure 4:**
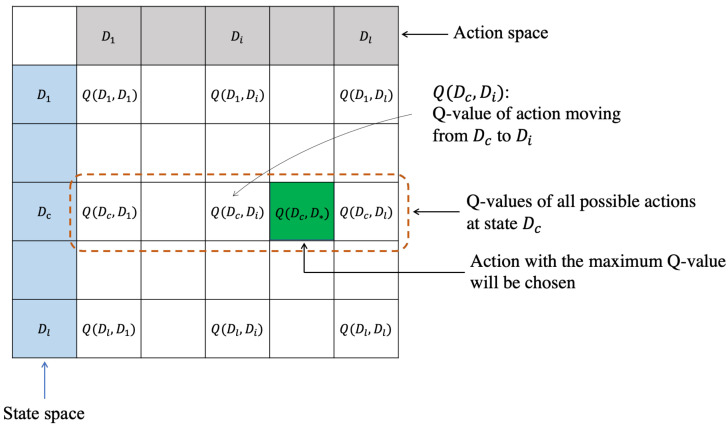
An illustration of the Q-table.

In our Q-learning-based model, the network is considered the environment while the MC is the agent. A state is defined by the current charging location of the MC, and an action is a move to the next charging location. Each MC maintains its own Q-table, which is a two-dimensional array. Each row represents a state, and each column represents an action. An item $Q(D_{j},D_{i})$ in the *j*-th row and *i*-th column represents the Q-value corresponding to the action when the MC moves from the current charging square $D_{j}$ to the next charging location $D_{i}$. Figure 4 shows an illustration of our Q-table. In the figure, the gray row represents the Q-values concerning all possible actions when the MC stays at the charging location $D_{c}$. The green cell depicts the maximum Q-value regarding the next charging location.

Let $D_{c}$ be the current charging location and $D_{i}$ be an arbitrary charging location, then the Q-value of action moving from $D_{c}$ to $D_{i}$ is iteratively updated by using the Bellman equation as follows:

(4) $$Q\left(D_{c},D_{i}\right)\leftarrow Q\left(D_{c},D_{i}\right)+\alpha \left(r\left(D_{i}\right)+\gamma \underset{1\leq j\leq l}{max}Q\left(D_{i},D_{j}\right)-Q\left(D_{c},D_{i}\right)\right).$$

The equation ’s right side consists of two elements, including the current Q-value and the temporal difference. The temporal difference measures the gap between the estimated target, i.e., $r\left(D_{i}\right)+\gamma \underset{1\leq j\leq l}{max}Q\left(D_{i},D_{j}\right)$, and the old Q-value, i.e., $Q\left(D_{c},D_{i}\right)$. α and γ are two hyper-parameters whose names are learning rate and discount factor, respectively. $r(D_{i})$ is our proposed reward function, which will be detailed in Section 4.5.

In the following, we first describe our algorithms to determine the optimal charging time and the safety energy level in Section 4.3 and Section 4.4. Then, we present the details of the reward function and the mechanism for updating the Q-table in Section 4.5 and Section 4.6.

### 4.3. Charging Time Determination

We aim to design a charging strategy so that the number of sensors reaching a safe energy level is as big as possible after each charging round. Here, the safe energy level means the energy amount that is sufficiently greater than $E_{th}$. We define the safe energy level, $E_{sf}$, as

(5) $$E_{sf}=E_{th}+\theta E_{max},$$

where $E_{max}$ is the maximum energy capacity of the sensors. θ is an adaptive parameter, named safe energy factor, which is determined by Fuzzy logic. The algorithm determining θ algorithm will be described in Section 4.4.

A sensor node has the *critical status* if its remaining energy is smaller than to $E_{sf}$. The sensor with a critical status is named as *critical sensor*. Otherwise, a sensor node is called a *normal sensor*. For each charging location $D_{i}$ (1≤i≤l), we want to determine the optimal charging time $T_{i}$ to minimize the number of critical sensors.

We adopt the multi-nodes charging model, in which the MC can simultaneously charge to all sensors. We leverage the charging model proposed in [31], which has been widely used in most research in the WRSN-related studies. This model follows the RF-power harvesting. The authors in [31] have performed experiments to verify the model in the real environment. Following the experiment results shown in the paper, the charging energy is about $12\times 10^{-4}$ W when the distance is 1.6 m. According to [31], the per second energy that a sensor $S_{j}$ is charged when the MC stays at $D_{i}$ is given by

(6) $$p_{j}^{i}=\frac{\lambda }{{\left(d\left(S_{j},D_{i}\right)+\beta \right)}^{2}},$$

where λ and β are known constants decided by the hardware of the charger and receiver. $d(S_{j},D_{i})$ is the Euclidean distance between $S_{j}$ and $D_{i}$. We denote $e_{j}$ as the energy consumption rate of $S_{j}$, which is estimated by the MC. Suppose that the MC charges $S_{j}$ at $D_{i}$, we denote the remaining energy of $S_{j}$ when the charging process starts and finishes as $E_{j}$ and $E_{j}^{{}^{\prime }}$, then $E_{j}^{{}^{\prime }}=E_{j}+\left(p_{j}^{i}-e_{j}\right)\times T_{i}$. At the charging location $D_{i}$, we call $p_{j}^{i}-e_{j}$ the *energy gain* of $S_{j}$. The remaining energy of $S_{j}$ will increase if its energy gain is positive and decreases otherwise. Note that the energy of $S_{j}$ equals the safety energy level, if the charging time equals to $\frac{E_{sf}-E_{a_{j}}}{p_{a_{j}}^{i}-e_{a_{j}}}$, which is named as the *safety charging time* of $S_{j}$ with respect to the charging location $D_{i}$ and denoted as $\Delta_{j}^{i}$. The sensors can be classified into four groups. The first and second ones contain normal sensors with positive energy gain and critical sensors with negative energy gain, respectively. The third and fourth groups contain normal sensors with negative energy gain and critical sensors with positive energy gain, respectively. Obviously, the first and second groups’ sensors do not change their status no matter how long the MC charges at $D_{i}$. In contrast, a sensor $S_{j}$ in the third group will fall into the critical status, and a sensor in the four groups can alleviate the critical status, if the charging time $T_{i}$ is greater than or equals to its safety charging time, i.e., $T_{i}>\Delta_{j}^{i}$. Hereafter, we call the sensors in the third group *negative normal sensors* and the sensors in the fourth group *positive critical sensors*. Let $\epsilon_{1},\epsilon_{2}$ denote the number of sensors belonging to the third and fourth groups whose status changes after being charged (from critical to normal and vice versa). It is worth noting that the greater the value of $T_{i}$, the greater $\epsilon_{1}$, and the greater $\epsilon_{2}$, also. Our objective is to determine the optimal value of $T_{i}$ to maximize $\epsilon_{1}-\epsilon_{2}$. This goal can be achieved by using the following algorithm.

- First, we calculate the safety energy charging time of all negative normal sensors (denoted as $\Delta_{a_{1}}^{i},\ldots ,\Delta_{a_{u}}^{i}$) and positive critical sensors (denoted as $\Delta_{b_{1}}^{i},\ldots ,\Delta_{b_{v}}^{i}$).
- We then combine the values of $\Delta_{a_{1}}^{i},\ldots ,\Delta_{a_{u}}^{i}$ and $\Delta_{b_{1}}^{i},\ldots ,\Delta_{b_{v}}^{i}$ into an array denoted as $\Delta_{c_{1}}^{i},\ldots ,\Delta_{c_{u+v}}^{i}$, where $\Delta_{c_{1}}^{i},\ldots ,\Delta_{c_{u+v}}^{i}$ have been sorted by decreasing order (i.e., $\Delta_{c_{1}}^{i}\geq \Delta_{c_{2}}^{i}\geq \ldots \geq \Delta_{c_{u+v}}^{i}$). We have an important observation that the value of ($\epsilon_{1}-\epsilon_{2}$) does not change when $T_{i}$ varies in the range from $\Delta_{c_{p}}^{i}$ to $\Delta_{c_{p+1}}^{i}$ (1≤p≤u+v). Therefore, the optimal value of $T_{i}$ can be easily determined by brute force search over $\Delta_{c_{1}}^{i},\ldots ,\Delta_{c_{u+v}}^{i}$.

### 4.4. Fuzzy Logic-Based Safe Energy Level Determination

#### 4.4.1. Motivation

We observe that $E_{sf}$ contributes significantly to the algorithm’s performance. When $E_{sf}$’s value is small, the MC’s maximum energy amount, charging to the sensors, is also small. Accordingly, after being charged, the sensor’s battery may quickly go below the threshold. On the contrary, if $E_{sf}$ is too large, the MC needs to spend a long time at every charging point. Consequently, the sensors far from the charging points may run out of energy while waiting for their turn. To this end, we leverage Fuzzy logic to adjust $E_{sf}$’s value adaptively. In the following, we first analyze factors that affect $E_{sf}$. We then describe the proposed algorithm. Remember that according to Equation (5), $E_{sf}$ is determined by $E_{sf}=E_{th}+\theta E_{max}$. Therefore, below, we will show how to adjust the value of θ adaptively.

When the MC stays at a charging point, the nearby sensors receive a more significant energy amount than the faraway sensors. Therefore, each near sensor’s charging amount is likely more significant than its energy consumption for sensing and communication. Consequently, the nearby ones tend to increase their battery level gradually. On the contrary, the faraway sensors tend to decrease due to the energy consumption for sensing and communication tasks. It is expected that the MC should spend a longer time at charging points where sensors far from it do not encounter critical situations (i.e., having low residual energy or high energy consumption rate). Based on this observation, our algorithm is designed so that $E_{sf}$ tends to receive a small value in the following cases.

- The residual energy of all sensors is small.
- Many sensors need to be charged.

We propose a Fuzzy logic-based $E_{sf}$ determination algorithm, which utilizes the following two variables. The first one is the minimum residual energy of all sensors $E_{min}$. The second is the number of charging requests, denoted as $L_{r}$.

The details of the algorithm is presented in Algorithm 1.

#### 4.4.2. Fuzzification

**Figure 5:**
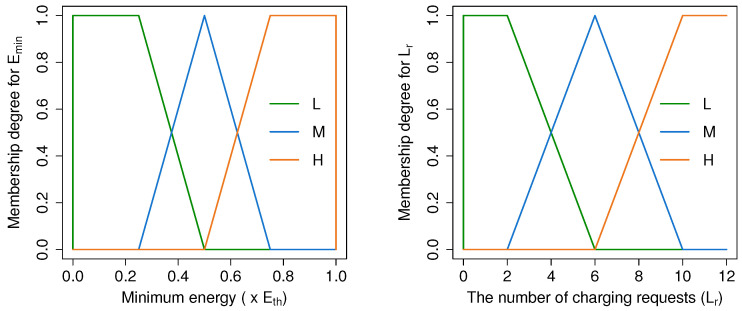
Fuzzy input membership functions.

**Figure 6:**
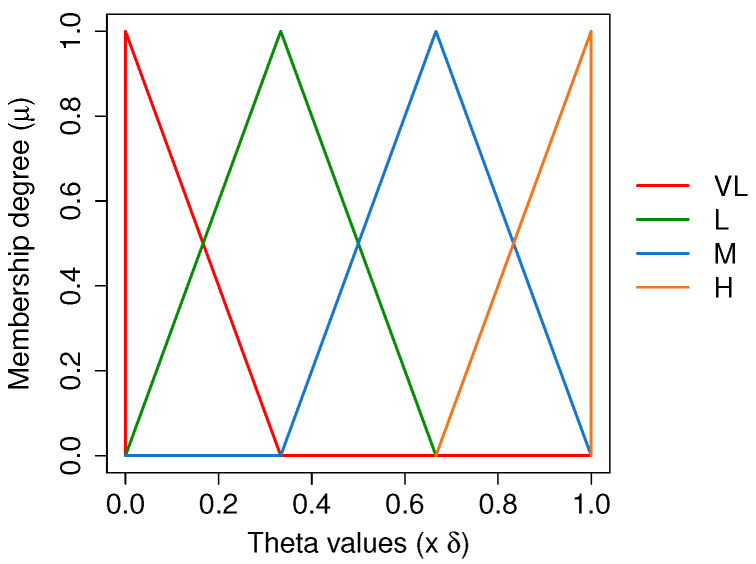
Fuzzy output membership functions.

With the two variables $L_{r}$ and $E_{min}$, we denote the output as the value of θ. Each input is mapped into three linguistic variables that are *low, medium*, and *high*. Meanwhile, the output is mapped into five ones, namely *very low, low, medium*, and *high*. We leverage the triangular and trapezoidal fuzzy numbers, whose formulas are given below:

(7) $$Triangular\left(x,a,b,c\right)=\left\{\begin{array}{ccc} 0 & , & x\leq a\\ \frac{x-a}{b-a} & , & a\leq x\leq b\\ \frac{c-x}{c-b} & , & b\leq x\leq c\\ 0 & , & c\leq x \end{array}\right.$$

(8) $$Trapezoidal\left(x,a,b,c,d\right)=\left\{\begin{array}{ccc} 0 & , & x\leq a\\ \frac{x-a}{b-a} & , & a\leq x\leq b\\ 1 & , & b\leq x\leq c\\ \frac{d-x}{d-c} & , & c\leq x\leq d\\ 0 & , & d\leq x \end{array},\right.$$

where *x* is the crisp input, and a,b,c,d are membership function ranges of the fuzzy variables. The values of a,b,c,d are represented in Table 2 and Table 3. Figure 5 and Figure 6 depict the Fuzzy membership functions of the input and the output variables, respectively.

#### 4.4.3. Fuzzy Controller

There are two input variables; each is converted to three fuzzy sets, so we have a total of $3^{2}=9$ rules in the *Knowledge Base*, which are listed in Table 4. The rules are designed to reflect the observation described in Section 4.4.1. Our rules have the form of “**IF** ($L_{r}$ is *A*) **AND** ($E_{min}$ is *B*) **THEN** (θ is *D*)”, in which A,B obtain the values of *low, medium*, or *high*, and *D* is either *very low, low, medium*, or *high*. For the ease of presentation, we use the following notations: *VL = very low, L = low, M = medium, H = high*. As the Fuzzy rules are based on the **AND** operator, the output membership degree is defined by

(9) $$\mu_{R_{i}}=\min\left\{\mu_{A}\left(L_{r}\right),\mu_{B}\left(E_{min}\right)\right\},\forall i=1,\ldots ,9.$$

#### 4.4.4. Defuzzification

After the *Inference Engine* determines the output’s membership degree on fuzzy sets (by using Equation (9)), the fuzzy set with the highest membership degree is considered the output variable. Finally, we utilize the *CoG* function to calculate the crisp value of the output’s fuzzy set.

Let us consider an example where $L_{r}=3,E_{min}=3$, and $E_{th}=4$. First, the fuzzy inputs of the two variables $L_{r}$ and $E_{min}$ are calculated using Equations (7) and (8), as presented in Table 5. Then, we use Equation (9) to calculate the output membership degree of each fuzzy rule. For example, the fuzzy output for rule $R_{1}$ is given by

$$\begin{array}{ccc} \mu_{R_{1}} & = & \min(\mu_{L}\left(L_{r}\right),\mu_{L}\left(E_{min}\right))\\ & = & \min(0,0)=0. \end{array}$$

The values of all the fuzzy rules are shown in Table 6. From Table 6, it can be seen that rule 3 achieves the highest membership degree. Therefore, the output variable θ obtains the linguistic value of *low(L)*. Finally, we use the *CoG* function to convert the output’s linguistic variable into a crisp value. According to Equation (3), the *CoG* of the output linguistic variable *low(L)* is defined by

(10) $$CoG_{L}=\frac{\int_{0}^{\delta }\theta \mu_{L}\left(\theta \right)d\theta }{\int_{0}^{\delta }\mu_{L}\left(\theta \right)d\theta },$$

where $\mu_{L}$ is the membership function of *low(L)*. Following the definition of the output membership function represented in Table 3, we have

(11) $$\mu_{L}\left(\theta \right)=\left\{\begin{array}{ccc} \frac{3}{\delta }\theta & , & 0\leq \theta \leq \frac{1}{3}\delta \\ 2-\frac{3}{\delta }\theta & , & \frac{1}{3}\delta \leq \theta \leq \frac{2}{3}\delta \\ 0 & , & \frac{2}{3}\delta \leq \theta \leq \delta . \end{array}\right.$$

where $\delta =\left(0.1-2\frac{E_{th}}{E_{max}}\right)$. By substituting Equation (10) into Equation (11), we obtain the value of $CoG_{M}$ as $\frac{1}{3}\delta$. It means that with $L_{r}=3,E_{min}=3$, and $E_{th}=4$, the value of θ is $\frac{1}{3}\delta$, thus the *safe energy level* $E_{sf}$ is given by $E_{sf}=E_{th}+\frac{1}{3}\delta E_{max}$.
**Algorithm 1:** Fuzzy Logic-based θ determination
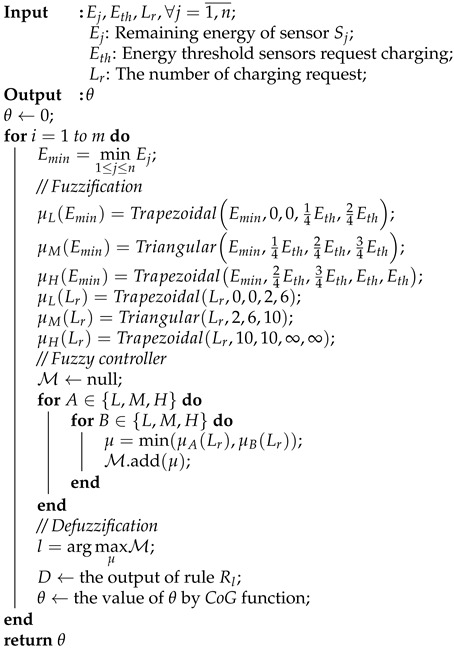


### 4.5. Reward Function

Our objective is to maximize the network lifetime. To achieve this goal, we need to guarantee that every target is monitored by at least one sensor. Note that a target is considered to be monitored by a sensor if it is covered by a sensor (i.e., remaining within the sensor’s sensing range) and the sensor is connected to the base station (i.e., a routing path exists between the sensor and the base station). Hence, the next charging location of MC should be selected to prioritize the following sensors:

- The sensors with either high energy consumption rate or low level of remaining energy;
- The sensors either cover many targets or participate in many routing paths from target-covering sensors to the base station.

We emphasize that our design can be applied to any routing protocol. However, in the experiments in Section 5, we adopt the geographic greedy routing protocol [32]. The geographic greedy routing protocol is widely accepted in WSNs due to its simplicity and efficiency. In this routing protocol, each node chooses the next hop to be the neighboring node closest to the destination. It is worth noting that in geographic routing, it is usually assumed that every node knows its location, and the location of its one-hop neighbors. This assumption can be realized by using positioning services [33] and the neighbor notification packets, respectively. Besides, the source node knows the position of the destination node. This assumption is legitimate in geographic routing [34,35,36,37,38,39,40,41,42]. To determine the next node, the current node only needs to look up in its neighbor table and find the one with the smallest distance to the destination. The computational complexity for determining such the next node is only O(m), where *m* is the number of the current node’s one-hop neighbors. For each sensor $S_{j}$, we define an *energy severity index* (denoted as $\xi_{j}$) which is calculated by the ratio of $S_{j}$’s energy consumption rate $e_{j}$ to its remaining energy $E_{j}$ ($\xi_{j}=\frac{e_{j}}{E_{j}}$). We can see that the higher the energy severity index, the more critical the sensor is. Hence, the MC should charge a larger amount of energy to the sensor. We name a *priority index* for each sensor, which indicates the sensor’s importance in covering targets and transferring sensory data to the base station. Specifically, a sensor $S_{j}$ has the priority index $w_{j}$, defined as a sum of its covered target number and the routing-path number. The routing path is from a target-covering sensor to the base station through $S_{j}$. Similarly, the priority index is proportional to the significant effects on the target coverage and connectivity. The sensor with a higher priority index needs more energy from the MC than a lower-index one does.

We reflect the observation mentioned above in the design of the reward function, which considers three factors *energy factor*, *sensor priority factor*, and *target monitoring factor*. The first two factors depict the relationship between the sensors’ energy severity indexes and priority indexes with the energy they will be charged. The last factor estimates the number of monitored targets. We denote a charging location $D_{i}$’s energy factor, sensor priority factor, and target monitoring factor as $E\left(D_{i}\right),P\left(D_{i}\right)$, and $T(D_{i})$, respectively. The first two factors are designed based on a well-known fact that given two sorted number sets $a_{1}\geq a_{2}\geq \ldots \geq a_{n}$ and $b_{1}\geq b_{2}\geq \ldots \geq b_{n}$. Let $\pi_{1}$ and $\pi_{2}$ be two permutations of {1,2,…,n}, then $\sum_{i=1}^{n}a_{\pi_{1}\left(i\right)}b_{\pi_{2}\left(i\right)}$ attains the greatest value when $\pi_{1}=\pi_{2}=\left\{1,\ldots ,n\right\}$. Intuitively, $\sum_{i=1}^{n}a_{\pi_{1}\left(i\right)}b_{\pi_{2}\left(i\right)}$ is maximized when $a_{\pi_{1}\left(i\right)}$ is proportional with $b_{\pi_{2}\left(i\right)}$.

First, the energy factor $E(D_{i})$ is defined by $\sum_{j=1}^{n}p_{j}^{i}\times \xi_{j}$, which sums up the products of energy charged to the sensors and their severity indexes. According to the fact mentioned above, $E(D_{i})$ tends to achieve the maximal value when $p_{j}^{i}$ is proportional with $\xi_{j}$. It means that $E(D_{i})$ will increase if we increase the value of $p_{j}^{i}$ for sensors with a large value of $e_{j}$ and a small value of $E_{j}$. In other words, our algorithm encourages charging more to sensors with a large energy consumption rate and small remaining energy.

The priority factor $P(D_{i})$ is calculated by $\sum_{j=1}^{n}w_{j}p_{j}^{i}$, where $w_{j}$ is the priority of sensor $S_{j}$. Obviously, the second factor is maximized if $w_{j}$ is proportional with $p_{j}^{i}$. Therefore, our algorithm will prioritize the sensors that play important roles in guaranteeing the network’s target coverage and connectivity.

Third, the target monitoring factor $T\left(D_{i}\right)=\frac{t_{i}}{m}$ depicts the ratio of the number of targets that are monitored (i.e., $t_{i}$) to the total number of the targets (i.e., *m*). By including this factor, our algorithm will choose the next charging location that maximizes the number of monitored targets.

The reward of a charging location $D_{i}$ (denoted by $r(D_{i})$) is the normalized sum of the three factors. $r(D_{i})$ can be calculated as follows.

(12) $$r\left(D_{i}\right)=\frac{\sum_{j=1}^{n}\frac{1}{E_{j}}p_{j}^{i}e_{j}}{\sum_{k=1}^{l}\sum_{j=1}^{n}\frac{1}{E_{j}}p_{j}^{k}e_{j}}+\frac{\sum_{j=1}^{n}w_{j}p_{j}^{i}}{\sum_{k=1}^{l}\sum_{j=1}^{n}w_{j}p_{j}^{k}}+\frac{t_{i}}{\sum_{k=1}^{l}t_{k}}.$$

### 4.6. Q Table Update

To determine the optimal charging time and calculate the charging locations’ Q-value, the MC needs information about the sensors’ remaining energy and energy consumption rate.

Since the base station gathers and transfers all sensors’ remaining energy information to the MC periodically, the MC can estimate every sensor’s energy consumption rate based on the received information. In this work, we leverage a simple weighted averaging method to estimate the energy consumption rate. The energy consumption rate of sensor $S_{j}$ is defined by the average of its energy consumption rate at *L* timings in the past weighted by the corresponding time,

$$e_{j}=\frac{\sum_{k=1}^{L}e_{j}^{k}\times t_{k}}{\sum_{k=1}^{L}t_{k}},$$

where $e_{j}^{k}$ is the energy consumption rate of $e_{j}$ at timing $t_{k}$ in the past.

The more frequently the sensors update the information to the base station, the more accurately the MC can estimate the sensors’ energy consumption rate. However, sending such information too often may consume significant energy of sensors. Therefore, in our algorithm, the sensors only update their remaining energy in the following scenarios.

## 5. Performance Evaluation

We compare the performance of Fuzzy Q-charging with the most relevant four existing algorithms. The first one is INMA [16], in which the MC determines the next sensor to charge based on factors, including the residual energy of sensors and the distance from sensors to the MC. The next charging sensor is chosen to minimize the number of other requesting nodes that may suffer from energy depletion. The second one is GSA [17]. At each charging round in GSA, the MC uses the gravitational search algorithm to determine a near-optimal charging order to fulfill all charging requests. In both INMA and GSA, the MC always charges to the maximum battery capacity of the sensor. The third comparison benchmark is RMP-RL [15]. RMP-RL uses the Deep Q-learning technique to determine the charging path of the MC. The objective is to minimize the number of dead sensor nodes and the moving distance of MC. The last comparison benchmark is our previous work, namely Q-charging [30]. Q-charging leverages Q-learning to determine the next charging location. However, different from Fuzzy Q-charging, Q-learning tries to maximize the number of sensors being charged to a predefined energy level. Besides, we also measure the network lifetime when there is no charging scheme is applied. Hereafter, we call this option a “no-charging” scheme.

We conduct two experiments, among which the first complements the other. The first experiment investigates the impact of parameters γ and α on the performance of our proposal. Based on the first experiment results, we determine the optimal values of γ and α. They are used in the second experiment, which compares Fuzzy Q-charging performance to the existing works. The metrics of interest include the network lifetime and the number of non-monitored targets over time in the evaluation. In all experiments, the network area is fixed at the size of 100 m × 100 m. The sensors and targets were randomly scattered in the simulated region. The charging locations are positioned in the same place as the sensors. Each value plotted on the curves is the average obtained from 20 runs.

Regarding the charging model, we adopted the parameters proposed in [31,43]. The parameters have been verified by the experiments in [31,43]. More specifically, we set $\lambda =4.32\times 10^{-4}$, β=0.2316, $e_{MC}=10$ J/s, $e_{move}=0.01$ J/s. Moreover, the initial energy of sensors and MC are 10J and 100J, respectively. Each sensor has a battery capacity of 10J. In this simulation, we assume that sensors follow the Zigbee communication standard. We set the transmission range of sensors to 15 m. The reason is that based on the real experiment results reported in [44], the transmission becomes unreliable (i.e., the drop ratio becomes greater than 0) beyond 15 m. The velocity of the MC is 5 m/s. The average energy consumption rate of the sensors is estimated by the base station, as mentioned in Section 3. The parameters are summarized in Table 7.

### 5.1. Impacts of Parameters

This section studies the impacts of parameters α and γ on our proposed algorithm’s performance. Although we have conducted experiments with various settings, the results show similar trends. Therefore, we only present the results in a scenario with 300 sensors and 200 targets.

#### 5.1.1. Impacts of α

We vary the value of α from 0.3 to 0.8 and measure the network lifetime’s variation. The results are shown in Table 8. We can see the network lifetime enlarges significantly when α increases from 0.3 to 0.5. It dramatically drops when α reaches 0.6 and becomes stable. This phenomenon can be explained as follows. As shown in Equation (4), the new Q-value is calculated from the current Q-value, the reward, and the estimated maximal Q-value. α is the weight of the last two components, while 1−α is the weight of the first one. Intuitively, the current Q-value reflects the experience the agent has learned so far. Meanwhile, the reward and the estimated maximal Q-value can be seen as the knowledge the agent has just attained through the current action and the future prediction, respectively. When α is relatively small, e.g., less than 0.5, increasing α helps exploit the experience and the future forecast in making the decision, thus improving the goodness of the actions. However, when α is significantly large, the current experience and the future prediction dominate the Q-value. It means that the agent makes a decision primarily based on the current reward and future forecast and ignore all the experiences the agent has learned so far. The Q-learning now converges to the greedy approach. That is why the performance drops severely when α increases from 0.5 to 0.6 and becomes stable beyond that. From the experiment results, α should be moderate values around 0.4 and 0.5.

#### 5.1.2. Impacts of γ

Similarly, the impacts of γ is shown in Table 9. In this experiment, we set the value of α to 0.5. As can be observed, the network lifetime gradually decreases when γ increases. This is because γ is the weight of the predicted maximal Q-value in the future. The greater the γ, the more importantly the future prediction information contributes to the agent’s action. When γ is significantly small, the role of the future prediction (i.e., $Q_{max}$) in the Q-value is minor. Increasing γ helps agents exploit more future information in making action decisions, thus improving the decision’s goodness, thereby extending the network lifetime. However, when γ is significantly large, e.g., more than 0.6, increasing γ will eliminate the impacts of the current Q-value in making a decision. In other words, the agent tends to ignore all experiences learned so far and relies primarily on the future prediction. As the future prediction does not entirely correct, the performance of the Fuzzy Q-learning downgrades severely. From the experiment results, the optimal value of γ is from 0.4 to 0.5.

### 5.2. Comparison with Existing Algorithms

This section presents the comparison of our proposal to the existing ones. Following the previous observation, we set the values of α and γ to 0.5 and 0.4, respectively.

#### 5.2.1. Impacts of the Number of Sensors

**Figure 7:**
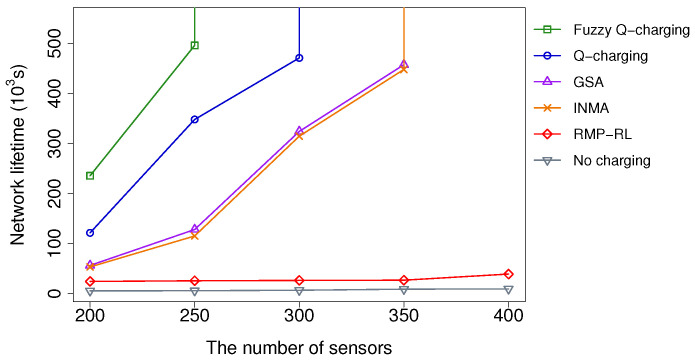
Network lifetime vs. the number of sensors.

Figure 7 depicts the network lifetime when the number of sensors varies from 200 to 400. In this experiment, the packets are generated randomly, with the probability of 0.2 packets in 1s; the target number is 150. The targets are randomly located in the network area. We can see that the network lifetime increases along with the increasing number of sensors in all algorithms due to each sensor’s traffic load has been reduced. However, Fuzzy Q-charging consistently outperforms the others. Ours can extend the network lifetime by at least 19.3 times. Moreover, the performance gaps between Fuzzy Q-charging and the others are proportional to the sensor number. When the number increases from 200 to 250, the gaps are small. However, the gaps dramatically change when reaching 300 sensors. Notably, when the number of sensors is 300, Fuzzy Q-charging extends the network lifetime infinitely, while Q-charging, INMA, and GSA can only attain a limited network lifetime. The reason is that when the number of sensors is small, the traffic imposed on each sensor is large. Therefore, the energy consumption rate of all sensors becomes immensely high. In all charging algorithms, the MC cannot charge to all sensors in time. That explains why the performance gap between the algorithms is insignificant with a small number of sensors. When the sensor number becomes sufficiently large, the energy consumption rate is slower. Fuzzy Q-charging favors the sensors with more essential roles in covering targets and transferring data to the base station. It can hence maintain the essential sensors’ lifetime and ensure all monitored targets. Other algorithms do not concurrently consider the target coverage and connectivity constraints. Therefore, the essential sensors may not be charged in time, causing some targets to be unmonitored.

Compared to Q-charging, i.e., the second-best charging algorithm, at the condition of fewer than 300 nodes, Fuzzy Q-learning’s network lifetime is 1.4 times better. In the case of 300 sensors, Fuzzy Q-learning’s network lifetime is infinite, while Q-learning’s one is only prolonged to about $500\times 10^{3}$ s. This results proves the effectiveness of our algorithm, which uses Fuzzy logic to automatically adjust the charging energy level. Concerning the two other algorithms, GSA, INMA, and RMP-RL, Fuzzy Q-learning improves the network lifetime to more than 4.3 times, at the condition of fewer than 300 nodes. Moreover, when the number of sensors reaches 300 nodes, GSA, INMA, and RMP-RL only prolong the network lifetime to less than about $448\times 10^{3}$ seconds, while that of Fuzzy Q-learning is infinite.

Among all the algorithms, RMP-RL shows the worst performance. The reason is that RMP-RL relies on the Deep reinforcement learning technique, which necessitates the training of a deep learning model. Unfortunately, a Deep reinforcement model typically takes a long time to converse. As a result, its early-stage performance is often poor, resulting in many dead nodes. This behavior is clearly demonstrated in Section 5.2.4.

#### 5.2.2. Impacts of the Number of Targets

**Figure 8:**
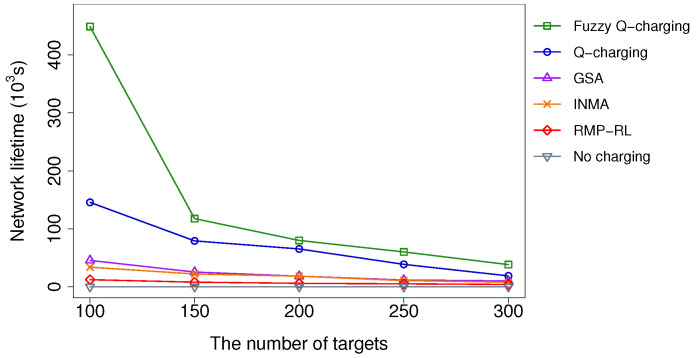
Network lifetime vs. the number of targets.

We evaluate the target number’s impact in a scenario with 300 nodes and the packet generation probability of 0.3. We investigate the network lifetime variation when the number of targets increases from 100 to 300. To do so, we first generate 300 targets randomly in the network region. After that, the number of targets is adjusted by subtracting 50, 100, 150, and 200 targets at random from the initial set. The results are presented in Figure 8. As shown, Fuzzy Q-charging performs much better than the other algorithms. As expected, as the number of targets increases, the network lifetime achieved by all algorithms drops. The reason for this is because when the target number increases, the volume of traffic on the sensors increases as well. When the number of targets is significantly large (i.e., more than 250), the energy consumption rate of sensors becomes too high. As a result, the MC is unable to charge the sensors in time. As a consequence, no algorithm can considerably increase the network lifetime.

When compared to the no-charge approach, Fuzzy Q-charging extends the network lifetime by more than 9.8 in all scenarios. Fuzzy Q-charging has average performance gaps of 6.3, 6.0, and 16.4 when compared to GSA, INMA, and RMP-RL, respectively. In the best case, the performance gaps of Fuzzy Q-charging to GSA, INMA, and RMP-RL are 12.6, 11.5, and 33.9, respectively. Compared to Q-charging, Fuzzy Q improves the network lifetime by 1.9 times in average and 3.1 times in the best case. The improvement of Fuzzy Q-charging is because Fuzzy Q-charging favors the sensors with more essential roles in covering targets and transferring data to the base station. It can hence maintain the essential sensors’ lifetime and ensure all monitored targets. Other algorithms do not concurrently consider the target coverage and connectivity constraints. Therefore, the essential sensors may not be charged in time, causing some targets to be unmonitored.

#### 5.2.3. Impacts of the Packet Generation Frequency

**Figure 9:**
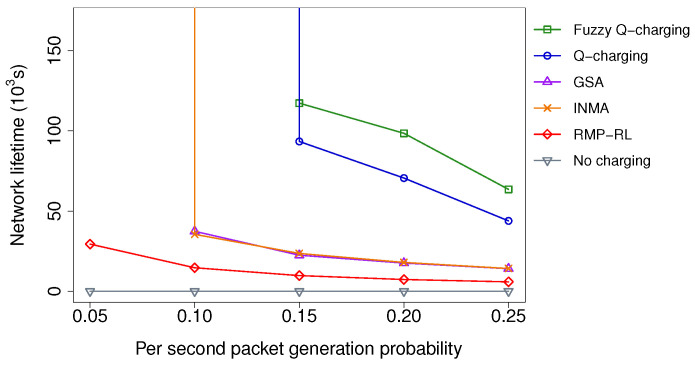
Network lifetime vs. the packet generation frequency.

Figure 9 shows the resulting impact of the packet generation probability on the network lifetime. In this experiment, the number of sensors and targets is set to 300 and 150, respectively. The location of the targets are generated randomly. In all algorithms, the network lifetime tends to decrease when the packet generation probability increases. When the probability is too large (i.e., being more than 0.25), all sensors’ energy consumption rate (especially sensors in the base station’s vicinity) becomes fast. Therefore, the sensors’ batteries exhaust quickly. In such a critical case, the difference between the algorithms is minor. We can see the improvement of Fuzzy Q-charging over the existing algorithms clearly under the condition of small packet generation probability. Notably, when the probability is 0.1, Fuzzy Q-charging prolongs the network lifetime infinitely, while the others cannot. When the probability is greater than 0.1, Fuzzy Q-charging’s network lifetime is 1.4 times more than Q-learning, 5.0 times more than INMA’s, 5.1 times more than GSA’s, and 21.1 times more than RMP-RL, on average. The performance gaps between Fuzzy Q-charging and the other algorithms decrease when the packet generation probability increases. Even when the probability is 0.25, the network lifetime’s ratio achieved by Fuzzy Q-charging is 1.4, 4.5, 4.5, and 10.8 times better than Q-charging, INMA, GSA, and RMP-RL, respectively.

In summary, we can conclude Fuzzy Q-charging outperforms the existing algorithms. Moreover, the performance gaps between Fuzzy Q-charging and the others increase when the number of sensors increases, the number of targets decreases, or the packet generation probability decreases.

#### 5.2.4. Non-Monitored Targets and Dead Sensors over Time

**Figure 10:**
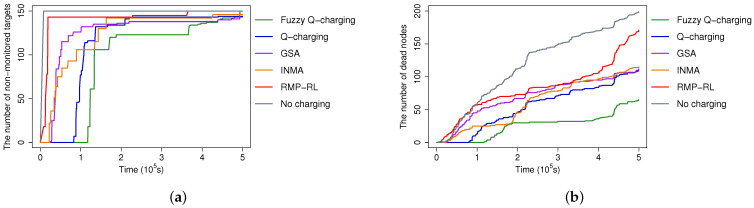
A comparison of non-monitored targets and dead sensors over time. (**a**) Non-monitored targets over time. (**b**) Dead sensors over time.

We present the number of non-monitored targets and the number of sensors over time caused by different algorithms in Figure 10a,b, respectively. In Figure 10b, when the time elapses, the number of sensors exhausting energy and becoming dead nodes increases. Accordingly, more targets become non-monitored, as shown in Figure 10a. Fuzzy Q-learning outperforms the other algorithms concerning both metrics. There is a huge gap between the performance of Fuzzy Q-learning and the others in Figure 10a. Fuzzy Q-charging with better charging strategies slows down the increase of non-monitored targets over time. Another interesting observation is that while the gaps between the number of dead sensors caused by using Fuzzy Q and that of INMA and GSA are relatively small (Figure 10b), the gaps concerning the number of non-monitored targets are huge (Figure 10a). The reason is INMA and GSA do not consider target coverage and connectivity constraints. Therefore, the next charging location is not optimized to prioritize the sensors with an essential role. Those sensors may be dead in INMA and GSA, leading to the targets being non-monitored. Meanwhile, in Fuzzy Q-charging, the charging location determination algorithm can identify the sensor nodes with a specific priority. Therefore, the dead sensors caused by Fuzzy Q-charging are the less important ones. In many cases, the death nodes may not affect or have minor impacts on the monitored targets.

## 6. Conclusions and Future Work

This paper addresses optimizing the MC’s charging schedule in WRSNs, which considers target coverage and connectivity constraints. Unlike the existing approaches, ours took into account the charging location and the charging time in the newly proposed Fuzzy Q-charging. Fuzzy Q-charging has an optimal charging time determination algorithm that relies on Fuzzy logic to adjust the energy charging level dynamically. The algorithm has been utilized at every charging point to maximize the number of alive sensors. Moreover, Fuzzy Q-charging uses Q-learning in an optimal charging scheme to maximize the target number. We have extensively evaluated Fuzzy Q-charging in comparison to the previous charging schemes in WRSNs. The evaluation results show that Fuzzy Q-charging outperforms the others. Specifically, Fuzzy Q-charging prolongs the network lifetime infinitely in certain conditions of the target and sensor numbers, while the other algorithms cannot. In other cases, Fuzzy Q-charging extends the network lifetime by 6.8 times on average and 33.9 times in the best case, compared to the existing algorithms. In the future, we plan to extend this work to handle the WRSNs with multiple mobile chargers.

## Figures and Tables

**Table 1:** List of notations.

Notation	Definition
$D_{i}$	The *i*-th charging location
$Q(D_{i},D_{j})$	Action value of the action moving from $D_{i}$ to $D_{j}$
$r(D_{i})$	Reward obtained after the MC moves to $D_{i}$
α	The learning rate of the Q-learning algorithm
γ	The discount factor of the Q-learning algorithm
$E_{th}$	The threshold for sending a charging request
$E_{sf}$	The safe charging level
θ	The safe energy factor
$E_{max}$	The maximum energy capacity of the sensors
$p_{j}^{i}$	The per second energy that a sensor $S_{j}$ is charged when the MC stays at $D_{i}$
$e_{j}$	Energy consumption rate of sensor $S_{j}$
$E_{j}$	Remaining energy of sensor $S_{j}$
$T_{i}$	The optimal charging time at $D_{i}$
$L_{r},E_{min}$	Fuzzy input variables
$w_{j}$	The priority index of $S_{j}$
$\xi_{j}$	The energy severity index of $S_{j}$, $\xi_{j}=\frac{e_{j}}{E_{j}}$.

**Table 2:** Input variables with their linguistic values and corresponding membership function.

Input Variable	Linguistic Value	Membership Function
$L_{r}$	*L*	$\left[0,0,2,6\right]$
	*M*	$\left[2,6,10\right]$
	*H*	$\left[6,10,\infty ,\infty \right]$
$E_{min}$	*L*	$\left[0,0,\frac{1}{4}E_{th},\frac{2}{4}E_{th}\right]$
	*M*	$\left[\frac{1}{4}E_{th},\frac{2}{4}E_{th},\frac{3}{4}E_{th}\right]$
	*H*	$\left[\frac{2}{4}E_{th},\frac{3}{4}E_{th},E_{th},E_{th}\right]$

**Table 3:** Output variable with its linguistic values and membership function, $\delta =\left(0.1-2\frac{E_{th}}{E_{max}}\right)$.

Output Variable	Linguistic Value	Membership Function
θ	*VL*	$\left[0,0,\frac{1}{3}\delta \right]$
	*L*	$\left[0,\frac{1}{3}\delta ,\frac{2}{3}\delta \right]$
	*M*	$\left[\frac{1}{3}\delta ,\frac{2}{3}\delta ,\delta \right]$
	H	$\left[\frac{2}{3}\delta ,\delta ,\delta \right]$

**Table 4:** Fuzzy rules for safe energy level determination.

$R_{\#}$	Input		Output
	$L_{r}$	$E_{\min}$	θ
1	*L*	*L*	*H*
2	*L*	*M*	*M*
3	*L*	*H*	*L*
4	*M*	*L*	*M*
5	*M*	*M*	*L*
6	*M*	*H*	*VL*
7	*H*	*L*	*L*
8	*H*	*M*	*VL*
9	*H*	*H*	*VL*

**Table 5:** Inputs of linguistic variables.

Input Variable	Membership Function	Value
$L_{r}$	$\mu_{L}$	0.75
	$\mu_{M}$	0.25
	$\mu_{H}$	0.00
$E_{min}$	$\mu_{L}$	0.00
	$\mu_{M}$	0.00
	$\mu_{H}$	1.00

**Table 6:** Fuzzy rules evaluation.

$R_{\#}$	$\mu_{R_{\#}}$	$R_{\#}$	$\mu_{R_{\#}}$	$R_{\#}$	$\mu_{R_{\#}}$
1	0.00	4	0.00	7	0.00
2	0.00	5	0.00	8	0.00
3	0.75	6	0.25	9	0.00

**Table 7:** System parameters.

Factor	Value
λ	$4.32\times 10^{-4}$
β	0.2316
Initial energy of the MC	100 J
Battery capacity of MC	500 J
The velocity of the MC	5 m/s
Initial energy of sensors	10 J
Battery capacity of sensors	10 J
$E_{th}$	4 J
Sensing range	7.5 m
Transmission range	15 m
Number of sensors	200~400
Number of targets	100~300
Per second packet generation probability	0.05~0.25

**Table 8:** The impact of α on the network lifetime.

α	0.3	0.4	0.5	0.6	0.7	0.8
Network lifetime ($10^{3}$ s)	235.042	246.601	246.268	243.493	243.719	244.183

**Table 9:** The impact of γ on the network lifetime.

γ	0.4	0.5	0.6	0.7	0.8
Network lifetime ($10^{3}$ s)	***∞***	***∞***	302.876	302.876	126.89

## Data Availability

Not applicable.
